# Prevalence of uterine synechia after abortion evacuation curettage

**DOI:** 10.1590/S1516-31802007000500002

**Published:** 2007-09-02

**Authors:** Adriana Salzani, Daniela Angerame Yela, José Roberto Erbolato Gabiatti, Aloísio José Bedone

**Keywords:** Hysteroscopy, Intrauterine diagnosis, Missed abortion, Incomplete abortion, Infertility, Histeroscopia, Diagnóstico pré-natal, Aborto retido, Aborto incompleto, Infertilidade

## Abstract

**CONTEXT AND OBJECTIVE:**

Intrauterine adhesion (IUA) is a possible complication of uterine curettage following abortion. Because IUA is an important cause of infertility, some investigators have been advocating its inclusion in the routine investigational workup after every abortion curettage procedure. The aim of this study was to evaluate the uterine cavity of patients subjected to abortion curettage, in order to ascertain the prevalence of IUA and its association with social and clinical factors.

**DESIGN AND SETTING:**

This was a cross-sectional study at the Human Reproduction Unit, Department of Obstetrics and Gynecology, Universidade Estadual de Campinas (Unicamp).

**METHODS:**

A total of 109 women were enrolled. The investigators searched the records of Unicamp's hospital for patients who had been subjected to uterine curettage following abortion. The hysteroscopy was performed 3 to 12 months after the curettage. The correlations between patients’ characteristics and the prevalence of IUA were assessed by means of chi-squared and Fisher's exact test calculations.

**RESULTS:**

The prevalence of IUA was 37.6%. The number of previous abortions and curettage procedures did not correlate with the presence of IUA. Most of the women (56.1%) presented IUA grade I.

**CONCLUSIONS:**

In the present study, 37.6% of the women subjected to curettage following abortion had IUA, which was mostly mucous and grade I. None of the demographic and clinic characteristics evaluated were found to be associated with IUA. From this study, there is no firm evidence to justify carrying out routine diagnostic hysteroscopy following abortion evacuation.

## RESUMO

**CONTEXTO E OBJETIVO:**

As sinéquias uterinas são complicações que podem ocorrer após curetagem uterina por aborto. Como se trata de causa importante de infertilidade, muitos autores têm indicado uma investigação rotineira após curetagens uterinas por aborto. O objetivo do estudo foi avaliar a cavidade uterina de pacientes submetidas a curetagem após aborto para detectar a prevalência das sinéquias e a possível associação com alguns fatores sociais e clínicos.

**TIPO DE ESTUDO E LOCAL:**

Estudo de corte transversal, realizado na Unidade de Reprodução Humana do Departamento de Ginecologia e Obstetrícia da Universidade Estadual de Campinas (Unicamp).

**MÉTODOS:**

109 mulheres foram submetidas a histeroscopia diagnóstica ambulatorial 3 a 12 meses após curetagem por aborto. Dados clínicos foram obtidos dos prontuários médicos. As correlações entre as características clínicas e a prevalência de sinéquias uterinas foram testadas através do cálculo do qui-quadrado e do teste exato de Fisher.

**RESULTADOS:**

A prevalência de sinéquias foi de 37,6%. O número de abortos anteriores e de curetagens não se correlacionou com a presença de sinéquias. A maioria das mulheres (56,1%) apresentou sinéquias grau I.

**CONCLUSÕES:**

Neste estudo, 37,6% das mulheres submetidas a curetagem por aborto apresentaram sinéquias, a maioria delas do tipo mucoso e de grau I. Nenhuma das características clínicas e demográficas avaliadas associou-se às sinéquias. Os resultados deste estudo não autorizam indicação rotineira de histeroscopia diagnóstica em pacientes submetidas a curetagem uterina por aborto.

## INTRODUCTION

Approximately 15% of all gestations will end as spontaneous abortion, which is defined as fetal death before 20 gestational weeks.^1^ Spontaneous resolution, or physiological elimination of the concept and its byproducts, is expected to happen in some of these cases, most notably if abortion occurred during the first gestational weeks. However, when gestational debris remains concealed inside the uterus, iatrogenic evacuation should be performed. Uterine curettage is the mainstay for uterine evacuation in several countries, and 243,998 curettage procedures were performed by the public health system in Brazil in the year 2004.^2^

Intrauterine adhesion (IUA) is a possible complication of curettage and its incidence has been reported to range from 15% to 40%.^3–9^ IUA is diagnosed by means of hysterography or hysteroscopy, and the different techniques used to diagnose the problem may account for the discrepancies in incidence rates. However, the frequency of IUA findings may vary according to several factors, such as the technique used to perform curettage, quality of preoperative and postoperative care, gestational age and clinical complications following abortion (particularly infections). The patient's constitutional characteristics probably also play a role.^10^

IUA causes infertility by tubal occlusion, distortions in the symmetry of the uterine cavity and damage to the basalis layer of the endometrium that predisposes towards abnormal placental implantation.^11^ Because of this, some investigators have been advocating the inclusion of hysteroscopy in the routine investigational workup after every abortion curettage procedure. Treatment for IUA is available, and consists of adhesiolysis through hysteroscopic intervention.^12^ However, it is debatable whether patients will derive any benefits from this policy following abortion, especially because the real incidence and prevalence rates of post-curettage IUA have not been definitely ascertained yet. Very unfortunately, the majority of studies^3–9^ addressing the incidence and prevalence of IUA have suffered from selection bias, as most of them only studied patients that had a clinical complications, most frequently infertility and menstrual abnormalities like amenorrhea and hypomenorrhea, derived from IUA.

## OBJECTIVE

Therefore, we performed this study to systematically evaluate the uterine cavity of patients who had been subjected to abortion curettage, three to twelve months after this procedure, in order to obtain direct and accurate measurements of IUA prevalence among these women.

## METHODS

For this cross-sectional study, a total of 109 consecutive women were enrolled. The sample size was calculated by taking a prevalence of 35%, significance level of 5%, β = 80% and sample error of 9%. This prevalence rate was obtained from a previous pilot study on 56 women.

The investigators searched the records of Unicamp's women's hospital, a tertiary public institution, for patients who had been subjected to conventional uterine curettage following abortion, and contacted these women to invite them to participate. A total of 278 women were contacted, but only 117 accepted entering the study. Eight patients were then excluded because they either became pregnant after their post-abortion curettage or had stenosis of the cervical ostium. Patients who accepted the invitation underwent a medical interview addressing clinical and epidemiological factors, and were then subjected to a complete pelvic examination followed by hysteroscopy. All the patients signed an informed consent statement and the study protocol had previously been approved by the local ethics committee. The hysteroscopy was performed three to twelve months after curettage.

The procedure was performed using a rigid 2.9 mm hysteroscope (Karl Storz, Germany). Carbon dioxide was used for uterine distension, by means of a hysteroinsufflator. Cervical dilatation was not performed and no analgesia was provided. The patients were discharged from hospital approximately one hour after the procedure was finished. The IUA severity was ascertained according to the classification of the European Society of Hysteroscopy^13^ and was recorded in the patients’ files.

The data were tabulated in electronic spreadsheets (Microsoft Excel^®^). The correlations between patients’ characteristics and prevalence of IUA were assessed by means of chi-squared and Fisher's exact test calculations. The correlations between patients’ characteristics and IUA grade were evaluated in the same way. All calculations were performed within 95% confidence intervals, using the SAS Statistical Software Package.

## RESULTS

The patients’ ages ranged from 18 to 45 years (mean: 28.4 years, standard deviation, SD = 7.53 years). Fifty-one percent of the patients were white. The mean gestational age at the time of the last abortion was 10.8 weeks (SD = 3.23 weeks). On average, the patients had had 1.31 abortions: 62.4% of these had been classified as incomplete and 37.6% as missed abortions. The incidence of infected abortion was 7.34% (data not shown). All the women presented normal uterine bleeding, except for depot medroxiprogesterone acetate users.

Forty-one patients (37.6%) were diagnosed with IUA. Most patients (73.4%) had had only one previous abortion, whereas 22.1% had two and the remainder had three previous abortions. The number of previous abortions did not correlate with presence of IUA (p = 0.57). Most patients (77.1%) had only been subjected to uterine curettage once, 18.3% twice and 4.6% three times. The number of previous curettage procedures was not associated with the presence of IUA (p = 0.69). The proportion of women whose last abortion was incomplete was 61.4%, but the type of their last abortion was not associated with IUA.

The vast majority (90.8%) of the women had been subjected to hysteroscopy within six months after their curettage, whereas only five (4.7%) had been examined up to nine months after curettage and another five (4.7%) up to 12 months after uterine evacuation. However, a significantly higher proportion of IUA was detected among the women who had been subjected to hysteroscopy between six and nine months after curettage (p = 0.01) (Table 1).

**Table 1.** Patients’ characteristics and intrauterine adhesions found in hysteroscopy

	Adhesion				
Number of gestations	No		yes		P*
	n	%	n	%	
1	21	(30.9)	15	(36.6)	
2	16	(23.5)	8	(19.5)	0.79
≥ 3	31	45.6)	18	(43.9)	
**Number of deliveries**					
0	30	(44.1)	17	(41.5)	
1	13	(19.1)	9	(22.0)	0.89
2	11	(16.2)	5	(12.2)	
≥ 3	14	(20.6)	10	(24.4)	
**Number of abortions**					
1	51	(75.0)	29	(70.7)	
2	15	(22.1)	9	(22.0)	0.57
3	2	(2.9)	3	(7.3)	
**Number of curettage procedures**					
1	54	(79.4)	30	(73.2)	
2	11	(16.2)	9	(22.0)	0.69
3	3	(4.4)	2	(4.9)	
**Type of abortion**					
Incomplete	42	(61.8)	25	(61.0)	
Missed	26	(38.2)	16	(39.0)	0.44
**Time after curettage**					
3-6 months	65	(95.6)	34	(82.9)	
6-9 months	0	0	5	(12.2)	0.01
9-12 months	3	(4.4)	2	(4.9)	

* Chi-squared or Fisher's exact test.

Only 13 women presented fibrous adhesions (11.9% of all the women) and twenty-eight had mucous adhesions. Most of them (51.2%) were isthmic and 31.7% were cornual (data not shown).

Among the patients diagnosed with IUA, most (56.1%) presented IUA grade I, whereas only 34.1% were categorized as grade II and 9.8% as grade III. The IUA grade was not affected by the number of gestations (p = 0.53), number of deliveries (p = 0.08), number of previous abortions (p = 0.74), number of curettage procedures (p = 0.55), type of abortion (p = 0.24) or length of time after curettage (p = 0.73) (Table 2).

**Table 2.** Patients’ characteristics and intrauterine adhesion (IUA) severity assessed by hysteroscopy

Number of gestations	IUA grade*			
	I	II	III	P†
1	10	4	1	
2	4	4	0	
≥ 3	9	6	3	0.53
**Number of deliveries**				
0	10	6	1	
1	7	2	0	
2	3	0	2	
≥ 3	3	6	1	0.08
**Number of abortions**				
1	17	10	2	
2	4	3	2	
3	2	1	0	0.74
**Number of curettage procedures**				
1	17	11	2	
2	4	3	2	
3	2	0	0	0.55
**Type of abortion**				
Incomplete	16	6	3	
Missed	7	8	1	0.24
**Time after curettage**				
3-6 months	20	11	3	
6-9 months	2	2	1	0.73
9-12 months	1	1	0	

* according to European Society of Hysteroscopy, 1989;

* Chi-squared or Fisher's exact test.

## DISCUSSION

In the present study, the prevalence of IUA following post-abortion curettage (37.6%) was higher than what was reported in most previous studies: 25.0%,^14^ 30.2%^5^ and 16.7%.^7^ Methodological differences may account for these discrepancies, most notably the diagnostic tools used for detecting IUA.

Another plausible explanation for the high prevalence of IUA in the present study is that even thin and filmy adhesions were included in the analysis. At the time of introducing the hysteroscope sheath, these adhesions were easily dissected. Importantly, the mean time elapsed between curettage and hysteroscopy in the present study was only 4.3 months, whereas it is expected that some adhesions may spontaneously remit over longer periods of time, especially mucous adhesions. To our knowledge, there is only one previous report with a similarly high prevalence of IUA prevalence, by Westendorp et al. (1998).^4^ These investigators found a 40% prevalence of IUA three months after curettage, but curettage procedures following term deliveries were also included in their report. Roge et al. (1996)^12^ reported that the proportion of filmy adhesions was higher when hysteroscopy was performed up to three months after curettage, as compared with hysteroscopy performed one year after the procedure. Similar findings have been reported by investigators who evaluated patients presenting sterility.^14^

The curettage technique is certainly dependent on the physician's skills. In the present study, most of the curettage procedures had been carried out by inexperienced first and second-year gynecology residents in a university hospital. The depth of abrasion is difficult to control^10^ and is related to the prevalence of post-curettage adhesions. Therefore, it is quite likely that the high prevalence of IUA in the present study might be a byproduct of the lack of expertise among the physicians performing the curettage.

There were no significant differences in IUA prevalence relating to the number of previous abortions among the patients of the present study, but such an association had been reported in previous investigations.^6^ However, like in previous reports, the IUA grade was proportionally higher among the women with three previous abortions. From a statistical standpoint, it is unfortunate that in the present sample only a few patients had three previous abortions and no patients had more than three previous abortions. This therefore prevented a more in-depth assessment of the relationship between number of abortions and prevalence of IUA.

Interestingly, there was no association between infected abortions and prevalence of IUA, thus corroborating previous studies.^7,10^ Similar prevalences of IUA were encountered among patients with previous incomplete or missed abortions. This finding was not unexpected, because some previous investigators had reported no differences relating to the nature of the abortion.^7^ Speculative and intuitive reasoning might lead to the belief that missed abortions could be related to IUA, because the dead concept remains in contact with the endometrium for a longer period, thereby eliciting fibroblastic activity and endometrial regeneration.^15^ However, the evidence has not given support to these theories.

The Brazilian Consensus on Gynecological Videoendoscopy was the motivation for the present investigators to carry out this study, based on the recommendation that hysteroscopy should be offered to all patients who are subjected to curettage for abortion or post-abortion evacuation, especially if these women desire a future pregnancy.^5^ The objective of this study was to determine the prevalence of IUA following post-abortion evacuation. However, from our results, no firm evidence was obtained to justify any modification in patient management.

## CONCLUSIONS

In the present study, 37.6% of the women subjected to post-abortion curettage had IUA, which was mostly mucous and grade I. None of the demographic and clinical characteristics evaluated were shown to be associated with IUA. Since the prevalence of severe IUA (grades II and III) is low, referral for hysteroscopy should be discussed extensively with the patient. From this study, there is no firm evidence to justify carrying out routine diagnostic hysteroscopy following abortion evacuation.
